# Arthroscopic treatment for femoroacetabular impingement syndrome (FAIS) in adolescents—5-year follow-up

**DOI:** 10.1093/jhps/hnab051

**Published:** 2021-07-03

**Authors:** Søren Winge, Sophie Winge, Otto Kraemer, Christian Dippmann, Per Hölmich

**Affiliations:** CPH Private Hospital, Rådhustorvet 4, Farum 3520, Denmark; Department of Vascular Surgery, Rigshospitalet, Blegdamsvej 9, Copenhagen Ø 2100, Denmark; Section of Sport Traumatology, Bispebjerg Hospital, Copenhagen University Hospital, Bispebjerg Bakke, Copenhagen 2400, Denmark; Sports Orthopedic Research Center-Copenhagen (SORC-C), Department of Orthopedic Surgery, Copenhagen University Hospital Hvidovre, Kettegaard alle 30, Hvidovre 2650, Denmark; Section of Sport Traumatology, Bispebjerg Hospital, Copenhagen University Hospital, Bispebjerg Bakke, Copenhagen 2400, Denmark

## Abstract

To report the minimum 5-year outcome after hip arthroscopy with labral repair in adolescents. From 2011 to 2014, 29 consecutive patients with a mean age 16.3 years (range 12.7–19.8 years) underwent hip arthroscopy treatment for femoroacetabular impingement syndrome. Patient-related outcome measures (PROMs) including modified Harris Hip Score (mHHS), Visual Analog Scale (VAS) for pain and Copenhagen Hip and Groin Outcome Score (HAGOS) were used preoperatively and at follow-up (FU). Percentage of patients achieving minimum clinically important difference (MCID) and substantial clinical benefit (SCB) for mHHS and HAGOS were determined. Mean FU was 6.7 years (range 5–9.6 years), and a 100% FU was accomplished. Significant improvements were seen for all PROMs at FU in patients not having a periacetabular osteotomy (PAO) with VAS pain score improving from mean 62 to 9, mHHS from 58 to 94 and HAGOS improved in all subgroups. For mHHS, SCB changes were achieved by 76% and MCID by 76% of the patients. Percentage of patients achieving MCID for HAGOS subgroups were 81% for pain, 67% for symptoms, 76% for physical function in daily living, 76% for physical function in sport and recreation, 81% for participation in physical activities and 81% for hip-related quality of life. Two patients had revision hip arthroscopy. PAO was later performed in three patients. The risk of further surgery with center edge (CE) bony edge (CEB) <30° was 42% and 0% with CEB ≥30°. Adolescents having hip arthroscopy with labral repair and resection of cam and pincer morphology achieve significant improvements at mean 6.7 years of FU. CEB < 30° increases the risk of further surgery.

## INTRODUCTION

Femoroacetabular impingement syndrome (FAIS) is defined as a motion-related clinical disorder of the hip with a triad of symptoms, clinical signs and imaging findings. Pincer morphology (excessive acetabular coverage of femoral head) and cam morphology (aspherical femoral head) are the two most common morphological variations of FAIS [1]. Among adolescents, FAIS is increasingly recognized as a source of hip pain [2–4]. There has been a dramatic increase in hip arthroscopy procedures also in the young age group below 30 years [5].

Recent work has suggested that FAIS can be formed in response to high activity levels during adolescence [3, 4, 6, 7]. It is presently unclear whether the resultant forces cause reactive bone formation at the head–neck junction or cause an alteration in physeal morphology, leading to cam morphology [8, 9]. There is a lack of theories explaining the etiology of pincer lesions [10].

Nevertheless, the presence of a cam deformity can contribute to the development of labral pathology and secondary osteoarthritis [11, 12].

Numerous studies have reported improved clinical outcomes after arthroscopic treatment [13–18].

However, there is currently limited medium- and long-term data reporting on the results of arthroscopic treatment for FAIS in adolescents [17, 18] and no previous study has selectively reported the outcome in adolescents using the Copenhagen Hip and Groin Outcome Score (HAGOS).

The purpose of this study was to report functional results, quality of life and the frequency of complications 5–9.6 years after arthroscopic treatment for FAIS among young people using validated patient-related outcome measures (PROMs) including modified ones Harris Hip Score (mHHS), HAGOS and Visual Analog Scale 0–100 (VAS) for pain. HAGOS is validated for use by young active patients [19].

We assumed that the clinical results would show significant improvement in all PROM parameters and that young people would experience clinically meaningful improvements after hip arthroscopy for FAIS and a low degree of complications.

## ETHICS

According to Danish law, no ethical approval is required when questionnaires are the only investigation form.

## MATERIALS AND METHODS

Inclusion criteria: Adolescents aged 10–19 years who have hip arthroscopy for FAIS [20]. They all had physiotherapy and activity modification for at least 5 months without sufficient effect.

Exclusion criteria: Prior surgery in the hip region. Dysplasia, Legg–Calvé–Perthes disease, slipped capital femoral epiphysis and arthritis of any kind based on X-ray and magnetic resonance imaging (MRI).

The mean time from onset of symptoms to surgery was 17 months (range 5–40 months). Only 28% (8/29) had a traumatic onset.

Between January 2011 and May 2014, 29 patients (18 females and 11 males) were included in the study. All patients were treated by the first author (SW). The average age was 16.3 years (range 12.7–19.8 years). All patients were preoperatively asked to complete a questionnaire consisting of age, sex, acute or gradual onset, duration of symptoms, mHHS, HAGOS and VAS for pain (0–100). VAS pain score was the patient’s average pain within the last 14 days.

Preoperative HAGOS was introduced later in the study and is only available in the last 21 patients. HAGOS has six items including pain, symptoms, physical function in daily living, physical function in sport and recreation, participation in physical activities and hip-related quality of life [19].

At follow-up (FU), the patients answered the same questionnaires via e-mail and reported complications and revision surgery.

The minimal clinical important difference (MCID) and the substantial clinical benefit (SCB and SCB change) were calculated using data for adolescents published by Nwachukwu (MCID ≥9.5 for mHHS, SCB ≥93.5 and SCB change >8.8 for mHHS) [21]. MCID and SCB can be considered complementary measures for defining a minimum and upper threshold for clinically significant outcomes [21].

MCID for HAGOS subgroups was calculated using ½ Standard Deviation (SD) of previously published HAGOS data by Thorborg [22].

It is not clear from most studies whether they report a center edge (CE) lateral sourcil (CES) or a CE-bone edge (CEB) and discrepancies exists with regard to the correct measurement of the CE angle [23, 24]. We measured the CEB.

### Radiographic findings

Standardized preoperative X-rays including anterior posterior (AP) pelvis and direct lateral view were obtained [25], and all patients had an MRI arthrogram to confirm labral pathology as well as other intraarticular and periarticular abnormalities.

**Fig. 1.**
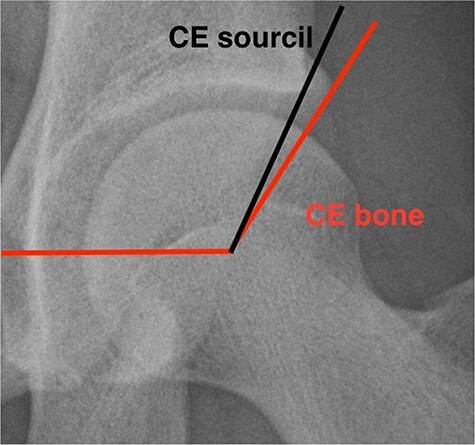
Center edge angle to sourcil and to bony edge (CE bone = CEB).

Using standardized X-rays, lateral CEB (Fig. 1) [23, 24], alpha angle (*α*), head–neck offset, cross-over sign, Tönnis angle and ischial spine sign were recorded as recommended by Tannast [25] and is presented in Table I. Cam type was defined as an alpha angle greater than 50° and pincer type was defined if X-rays showed a cross-over sign or a CEB >39° [25] (Table II).

**Table I.** Preoperative radiographic measurements (*n* = 29)

	*Mean*	*Range*
CEB (°)	31	(22−43)
TA (°)	6	(0−14)
Cross-over sign	25/29	
ISS	12/29	
*α* (°)	61	(42−89)
OS (mm)	7.6	(3.9−10.8)

CEB = center edge angle to bone, TA = Tönnis angle, ISS = ischial spine sign, *α* = alpha angle, OS = head–neck offset.

**Table II.** Distribution of impingement morphology

*Pathologies based on X-ray*	*n = 29*
Pincer only	7
Cam only	3
Pincer/cam combined	19

Cam = alpha angle >50°. Pincer = positive figure of 8 sign and/or CEB > 39°.

**Table III.** Patients with CEB <30° (*n* = 12)

						*VAS pain score (0–100)*
*Sex*	*Age (years)*	*CEB*	*Capsular closure*	*Additional surgery*	*mHHS at follow-up*	*at follow-up*
**♀**	15.3	24	Yes	PAO	82	7
**♀**	17.3	27	Yes	PAO	98	4
**♀**	17.5	22	Yes	PAO	100	2
**♀**	15.7	26	No		92	18
**♀**	14.6	22	Yes		100	0
**♀**	15.8	29	No	Revision	96	14
**♀**	18.4	29	Yes		100	1
**♀**	19.8	26	Yes	Revision	44	94
**♀**	14.3	27	Yes	−	96	4
♂	14.2	27	Yes		97	0
♂	15.4	28	No	−	100	0
♂	16.8	27	Yes	−	100	1
Mean	16.2	26			92^a^	12^a^

a Not significant from patients with CEB ≥30°.

The results for PAO patients are measured after PAO surgery. CEB = center edge angle to bone, PAO = periacetabular osteotomy, FU = follow-up, mHHS = modified Harris Hip Score, **♀ **= female, ♂= male.

### Surgical technique and findings

Hip arthroscopy was performed in the supine position under general anesthesia with fluoroscopic assistance. An intraportal capsulotomy was used in all patients. Surgical procedures were based on the preoperative and the intraoperative findings and included femoral osteoplasty in 27/29 patients and rim trimming to reduce cross-over sign or global pincer in 25/29 patients. Mean rim trimming was 2.7 mm (range 1–5 mm).

Cartilage injury in the acetabulum was recorded according to Beck’s classification [26].

In patients with a CEB angle within or close to the borderline zone between 20° and 25°, no rim trimming was performed. But the rim was debrided in order to increase the labral healing potential. Unstable cartilage was debrided to a stable rim. The labrum was repaired in all patients with a mean of 3.6 anchors.

At the end of the procedure, a full dynamic intraoperative range of motion test was performed to secure full decompression of the FAI. For the first six patients, we did not close the capsule; for the other patients, capsule closure was routinely used in all patients. The capsule was closed with 1 to 3 nonabsorbable sutures in 23 of the 29 patients. All included patients with CEB ≤25° had capsular closure.

The mean total surgical time (MTST) was 126 min. However, it was significantly reduced in the last 14 patients (MTST 110 min). Compared to the first 15 patients (MTST 140 min).

All patients were postoperatively referred to a standardized physiotherapy program for 3–4 months with 10 kg partial weight-bearing with crutches for 1–2 weeks, instant cycling on a stationary bike and circumduction exercises to reduce the risk of adhesions.

Surgical findings: All patients had a labral tear. Twenty patients had Beck grade 2 cartilage changes, with a wave sign of 3–5 mm, and five patients had Beck grade 3 changes with 3–5 mm of delamination.

### Statistics

Since HAGOS and mHHS do not show a normal distribution in healthy hips [22], non-parametric statistics with Wilkinson–Mann–Whitney U test were used with a significance level of 0.05.

## RESULTS

The mean FU time was 6.7 years (range 5–9.6 years), and a 100% FU was accomplished. The symptoms started at an average of 17 months before the operation (interval 5–40 months).

The mean VAS pain score decreased 53 points from 62 to 9 (*P* < 0.001) in patients not having a periacetabular osteotomy (PAO). The VAS pain score at FU was significantly lower in males (VAS 1) compared to females (VAS 14) (*P* < 0.05).

mHHS increased 35 points from 58 to 94 (*P* < 0.001) in patients not having a PAO. The 11 males had a mHHS mean score of 98 at FU, which was not significantly higher than the mean postoperative mHHS for the females, which was 91. The increase in mHHS and the decrease in VAS pain score were not significantly different between males and females. The VAS pain score, mHHS and HAGOS were not significantly different in patients having additional PAO surgery (Table IV). The majority of the patients (22/29) would repeat the surgery and 3/29 might repeat surgery.

**Table IV.** The mean FU scores and change for non-PAO and PAO patients

	*Non-PAO (n = 26)*		*PAO (n = 3)*	
	*FU*	*Change*	*FU^a^*	*Change^a^*
VAS pain score (0–100)	8	53	4	71
mHHS	94	34	93	42
HAGOS pain	95	35	94	51
HAGOS symptoms	88	24	81	29
HAGOS ADL	98	47	97	37
HAGOS sport & recreation	92	69	83	58
HAGOS participation in physical activities	88	68	88	83
HAGOS hip-related quality of life	87	59	82	58

a Not significant from non-PAO patients for all parameters.

PAO = periacetabular osteotomy, HAGOS = Copenhagen Hip and Groin Outcome Score, FU = follow-up, mHHS = modified Harris Hip Score.

**Fig. 2.**
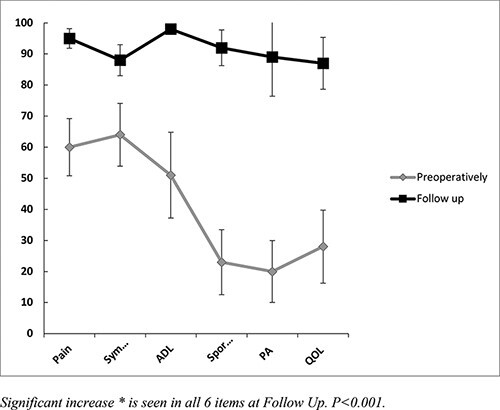
HAGOS with standard deviations in patients not having a PAO (*n* = 18). Significant increase is seen in all six items at follow-up. *P* < 0.001. ADL, physical function in daily living; Sport/rec, physical function in sport and recreation; PA, participation in physical activities; QOL, hip and/or groin-related quality of life.

In all HAGOS subgroups, the change in scores was highly significant for all items (Fig. 2).

MCID, SCB change and SCB end scores for mHHS are shown in Table V. MCID for HAGOS subgroups and the percentage reaching MCID are shown in Table VI.

**Table V.** Percentage (%) of patients reaching minimal clinical important difference (MCID), substantial clinical benefit (SCB) for modified Harris Hip Score (mHHS) at follow-up

*mHHS*	*%*
MCID (≥9.5)	76
SCB change (≥8.8)	76
SCB end score (>93.5)	66

PAO patients are considered not reaching MCID.

**Table VI.** Percentage (%) of patients reaching minimal clinical important difference (MCID) for Copenhagen Hip and Groin Outcome Score (HAGOS) subgroups at follow-up

*MCID*	*%*
HAGOS pain (MCID ≥ 9.1)	81
HAGOS symptoms (MCID ≥ 8.4)	67
HAGOS ADL (MCID ≥ 11.2)	76
HAGOS sport & recreation (MCID ≥ 9.9)	76
HAGOS participation in physical activities (MCID ≥ 12.1)	81
HAGOS hip-related quality of life (MCID ≥ 8.0)	81

PAO patients are considered not reaching MCID.

The mean CEB was 31° (range 22°–43°). We have only been able to retrospectively measure the CE sourcil in 15 patients since some of the original radiographs were lost during relocation of the hospital. The mean CE sourcil for these 15 patients was 26° (range 17°–39°).

CEB was <30° in 12 patients, and the results from this group are presented in Table III.

Three patients had a PAO at an average of 30 months after the hip arthroscopy at another hospital, and the FU results at an average of 50 months after the PAO were not significantly different from non-PAO patients (Table IV).

Two revision arthroscopies were performed with resection of capsulolabral adherences. One patient had erectile dysfunction for 4 months and recovered completely. This was probably related to the 2-h traction. One patient had an area of 5 × 5 cm on the lateral thigh with impaired sensation.

## DISCUSSION

We found highly significant improvements for both mHHS (58–94) and VAS pain score (62–9) at FU averaging 6.7 years.

Significant improvements for this age group have also been reported by others with shorter FU (1–3 years) [2, 21, 27–29] and mid-term and long-term FU [17, 18], indicating that the improvement after FAIS surgery in adolescents seems to remain at least for 5–10 years.

In these studies, labral repair was performed in 62–100% of patients, and capsular closure or capsular plication was performed in 82–100% of patients. This is similar to our study where all patients had a labral repair and 79% had capsular closure or capsular plication. The mid-term mHHS increase and the VAS pain score reduction seem to be higher in adolescents compared to adults [30, 31].

The Danish Hip Arthroscopy Registry (DHAR) study found better outcomes after FAIS surgery in patients <25 years compared to older patients [32], but other studies have not shown a significant difference in improvement between the two groups [13].

Contrary to the findings by Philipon [2], we found no significant difference in mHHS between males and females at FU, which could be due to a Type 2 error. In a major study from DHAR with patients between 9 and 79 years of age (mean 38), no difference found in PROMs between genders except for the Hip Sports Activity Scale (HSAS) 2 years after FAIS surgery [32].

We had two revision arthroscopies (6%), with the finding of capsulolabral adherences. We use circular motions of the hip [2], in our postoperative rehabilitation program, and this could explain the relatively low risk of capsulolabral adherence in our examination.

MCID for mHHS in FAIS surgery has been reported to be the same for adolescents and adults and the SCB (end score) to be significantly higher in adolescents [21].

The percentage of patients who obtained MCID for mHHS in this study (76%) is slightly smaller compared to other studies in juveniles [21, 29, 33] and higher compared to MCIDs found in adults after FAIS surgery [33].

SCB change was achieved by 76% of patients, and SCB end score was achieved by 66%, slighty less compared to other reported findings [21]. Less FU time (1 year) and FU rate (81%) can explain part of the difference in MCID and SCB scores.

The relatively low number of patients achieving SCB end score supports the finding that a very high postoperative mHHS is needed for adolescents in order to achieve SCB [21] and may explain why only 22/29 (76%) wanted to repeat the operation.

HAGOS improved significantly in all subgroups (*P* < 0.001), and the HAGOS at FU for all subgroups was higher than in studies of adults [14, 15, 22]. The percentage of patients achieving MCID for HAGOS in our study was higher than MCID values previously reported in a study of adult patients [22]. These differences can probably be explained partly by age at the time of surgery [32].

In other studies using HAGOS, capsular closure was only performed in <10% of surgeries [14, 15, 22], and in one study, labral repair was performed in <10% of the surgeries [14] compared to 100% labral repair and 79% capsular closure in our study. Both capsular closure and labral repair have been shown to improve results after arthroscopic FAIS surgery [34–36], and therefore, the difference in surgical technique may be part of the explanation for the difference in HAGOS results.

Of the seven patients who either might or would not repeat the surgery, only 1 was in the PAO and revision group. At FU, two patients not wanting surgery again had mHHS 100 and VAS 0 and 2 and one patient had mHHS 44 and VAS 94 but was very happy with the surgery and will repeat it, which suggests lack of coherence between the ‘issue of repeat surgery’ and PROMs for which we have no explanation.

Twelve patients had CEB <30° (Table III), indicating potential borderline dysplasia [22, 36]. The risk of having further surgery with CEB <30° was 42% and 0% in patients with CEB ≥30°. The CEB <30° patients had an end result, including PAO patients, not significantly different from the CEB ≥30° patients. The risk of having variable results with arthroscopic treatment for borderline dysplasia is well known [36]. Therefore, it is very important preoperatively to inform about the risk of further surgery if hip arthroscopy is considered as the primary treatment in patients with borderline dysplasia.

The complication rate in our study was 6%, which is comparable to other studies [27, 29]. The most common complications in adolescents seems to be traction-related and transient as one of the complications in our study [21, 27, 29].

The revision rate including PAOs was 17%, which is higher than reported reported by others (4.5–13%) in adolescents [2, 17, 27, 28], and all were females. A higher risk of revision in young females has been reported earlier [18]. Revision surgery and PAO surgery took place at an average of 36 months and longer FU seems to increase the risk of further surgery [18]. Less than 100% FU might lead to underreporting of revision surgery. Also, the use of CEB instead of CE sourcil in this study might have led to an underestimation of the ‘dysplasia’, thus a higher frequency of hip arthroscopy in potential borderline patients potentially increasing the risk of further PAO surgery.

It is still debated whether hip arthroscopy or PAO is the best treatment for borderline dysplasia patients [37, 38], and some Surgeons claim that there are good arguments for doing both arthroscopy and PAO in patients with dysplasia and therefore it can be discussed whether further PAO operations are a complication [39, 40].

In contrast to other reports [41, 42], our study indicates that hip arthroscopy can achieve successful results in patients with borderline dysplasia, who later have additional PAO surgery and support that borderline dysplastic girls have an increased risk of PAO after hip arthroscopy [43].

However, due to the very limited number of patients in our study, further research is needed.

Limitations of this study include a small sample size, absence of data from reexamination and postoperative radiographs. Furthermore, there was no control group.

The strength of this study includes a well-defined patient group with a 100% FU, minimum 5-year FU and the use of validated PROMs. All surgeries were performed by the same surgeon with uniform surgical technique and rehabilitation program for all cases.

## CONCLUSION

Adolescents with FAIS who have hip arthroscopy with labral repair and resection of cam and pincer morphology achieve significant clinical improvements and further PAO does not appear to affect the results for at least 5 years FU. CEB <30° increases the risk of further surgery.

## Data Availability

The data underlying this article will be shared on reasonable request to the corresponding author.
